# IKK/NF‐κB and ROS signal axes are involved in Tenacissoside H mediated inhibitory effects on LPS‐induced inflammatory osteolysis

**DOI:** 10.1111/cpr.13535

**Published:** 2023-08-08

**Authors:** Xiaoxiao Xie, Weiwei Chen, Minglian Xu, Junchun Chen, Tao Yang, Chaofeng Wang, Yuangang Su, Jinmin Zhao, Jiake Xu, Qian Liu

**Affiliations:** ^1^ Guangxi Key Laboratory of Regenerative Medicine, Orthopaedic Department The First Affiliated Hospital of Guangxi Medical University Nanning Guangxi China; ^2^ Collaborative Innovation Centre of Regenerative Medicine and Medical BioResource Development and Application Co‐constructed by the Province and Ministry, Life Sciences Institute, Guangxi Medical University Nanning Guangxi China; ^3^ School of Biomedical Sciences University of Western Australia Perth Western Australia Australia; ^4^ Faculty of Pharmaceutical Sciences, Shenzhen Institute of Advanced Technology, Chinese Academy of Sciences Shenzhen China

## Abstract

Periodontal disease and arthroplasty prosthesis loosening and destabilization are both associated with osteolysis, which is predominantly caused by abnormal bone resorption triggered by pro‐inflammatory cytokines. Osteoclasts (OCs) are critical players in the process. Concerns regarding the long‐term efficacy and side effects of current frontline therapies, however, remain. Alternative therapies are still required. The aim of this work was to investigate the involvement of Tenacissoside H (TDH) in RANKL‐mediated OC differentiation, as well as inflammatory osteolysis and associated processes. In vitro, bone marrow‐derived macrophages (BMMs) cultured with RANKL and M‐CSF were used to detect TDH in the differentiation and function of OCs. Real‐time quantitative PCR was used to measure the expression of specific genes and inflammatory factors in OCs. Western blot was used to identify NFATc1, IKK, NF‐κB, MAPK pathway, and oxidative stress‐related components. Finally, an LPS‐mediated calvarial osteolysis mouse model was employed to explore TDH's role in inflammatory osteolysis. The results showed that in vivo TDH inhibited the differentiation and resorption functions of OCs and down‐regulated the transcription of osteoclast‐specific genes, as well as *Il‐1β*, *Il‐6* and *Tnf‐α*. In addition, TDH inhibited the IKK and NF‐κB signalling pathways and down‐regulated the level of ROS. In vivo studies revealed that TDH improves the bone loss caused by LPS. TDH may be a new candidate or treatment for osteoclast‐associated inflammatory osteolytic disease.

## Graphical abstract

Tenacissoside H (TDH) is a monomer extracted from Marsdenia tenacissma and has been shown to be anti‐inflammatory and antioxidant. However, the role of TDH in skeletal diseases remains unclear. In this study, TDH was found to inhibit the IKK/NF‐κB signalling pathway, down‐regulate the expression of c‐FOS, NFATc1, CTSK, and ATP6v0d2, and suppress osteoclast differentiation and function. And TDH could inhibit lipopolysaccharide‐induced transcription of Il1‐β, Il‐6 and Tnf‐α, down‐regulate ROS levels, and In addition, in vivo studies showed that TDH significantly repaired LPS‐induced bone loss. TDH has clinical promise for antiosteoclast activity.

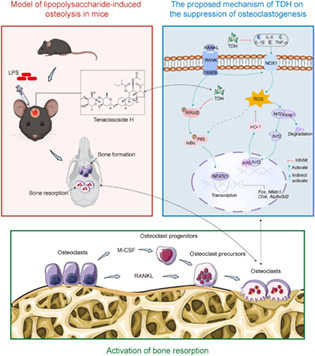

## 1 INTRODUCTION

Osteolysis is linked to a number of orthopaedic disorders, including metabolic bone disease, inflammatory arthritis, osteoporosis, osteonecrosis, and periprosthetic loosening.^1^ Acute inflammation is mostly caused by endogenous or exogenous unfavourable stimuli and can progress to chronic inflammation if not treated in time to preserve tissue homeostasis.^2^ Chronic inflammation inhibits osteoblasts (OBs) differentiation while activating OCs, and the equilibrium between bone repair and resorption becomes unstable.^3^ Despite the fact that immune cells, mesenchymal cells, and the environment are all involved in inflammatory osteolysis, OCs are essential actors in the process.^4^

OCs are the major bone‐resorbing cells generated from mononuclear macrophages.^5^ M‐CSF (macrophage colony‐stimulating factor) and receptor activator of nuclear factor kappa‐B ligand (RANKL) promote progenitor cells to develop into osteoclasts.^6^ Nuclear factor kappa‐B (NF‐κB) and mitogen‐activated protein kinase (MAPK) activation by RANKL leads to increased expression of Nuclear Factor of Activated T Cells 1 (NFATc1) in addition to their downstream targets, such as Cathepsin K (Ctsk) and ATPase H Transporting V0 Subunit D2 (Atp6v0d2).^7^ NFATc1 and c‐FOS are major transcription factors that regulate osteoclast differentiation, fusion and bone resorption.^8^ Atp6v0d2 plays a key role in pro‐osteoclast fusion,^9^ and Ctsk is a lysosomal cysteine protease that plays a key role in bone resorption.^10^

The primary cause of inflammatory osteolysis is abnormal bone resorption, which is exacerbated by pro‐inflammatory cytokines.^11^ Pro‐inflammatory cytokines like interleukin 1 (IL‐1β), interleukin 6 (IL‐6) and tumour necrosis factor (TNF‐α) promote osteoclasts to form on the bone surface.^12^ Lipopolysaccharide, a component of gram‐negative bacteria's outer membrane, acts as an inflammatory inducer, causing inflammatory cytokines to be released.^13^ It has been shown that following lipopolysaccharide (LPS) injection, the area of the eroded surface of the rat femur increases, as does the number of osteoclasts.^14^ Furthermore, reactive oxygen species (ROS) influences osteoclast development and function as a result of cellular metabolism and as a second messenger.^15^ Increased OC differentiation mediated by increased ROS in OCs results in severe inflammatory bone degeneration; in which, excessive ROS accumulation stimulates the Nrf2‐Keap1 pathway, where a large number of antioxidant enzymes are produced that safeguard osteoclasts against oxidative stress and minimize bone damage.^16^, ^17^

Some of the drugs currently used to treat bone resorption include estrogens, selective oestrogen receptor modulators, bisphosphonates, and denosumab; however, their use is limited in some circumstances due to side effects, contraindications, or cost.^15^ Chinese herbal medicines are clinically natural pharmaceuticals with proven clinical efficacy, and when used properly, they have significantly fewer adverse side effects than western medicine.^18^, ^19^ Tenacissoside H (TDH) is a monomeric extract of Marsdenia tenacissima that has been linked to a few pharmacological benefits, such as anti‐inflammatory, antioxidant, and anti‐tumour characteristics.^20^, ^21^, ^22^ TDH was found to inhibit IL‐1β, IL‐6 and TNF‐α expression and to mediate nitric oxide synthase, as well as inflammation and oxidative stress, thus modifying neurological recovery in mice with cerebral ischemia–reperfusion damage.^20^ TDH, on the other hand, has not been studied for osteolytic bone diseases.

In this study, we found that TDH inhibited the development of osteoclasts induced by RANKL via decreasing NF‐κB signalling and ROS generation, as well as LPS‐induced inflammatory factor release in vitro. Furthermore, in vivo studies revealed that TDH significantly repaired the bone loss caused by LPS. Thus, TDH may be beneficial in the prevention and control of inflammatory osteolysis.

## 2 MATERIALS AND METHODS

### 2.1 Reagents and antibodies

Chengdu Manstead Biotechnology Co. Ltd. (Chengdu, Sichuan, China) supplied TDH with an HPLC purity of ≥98.00%. R&D Systems, Inc. provided us with RANKL and M‐CSF. Gibco supplied foetal bovine serum (FBS), the fundamental medium of culture (α‐MEM), and penicillin–streptomycin solution (Thermo Fisher Scientific, Waltham, MA, USA). Kit‐8 (CCK‐8) was provided by Med Chem Express (MCE, Shanghai, China). Beyotime Biotechnology provided us with reagents for measuring alkaline phosphatase activity (Beyotime, Shanghai, China). Solarbio provided the solution of Alizarin Red S (1%, PH = 4.2) (Beijing, China). Cell Signalling Technology (CST) supplied the primary antibodies for p‐P38, P38, p‐JNK, JNK, p‐ERK, ERK, p‐P65, P65, IκBα, β‐actin, p‐IKKα/β, IKKα and IKKβ, as well as secondary antibodies (Danvers, MA, USA). Abcam supplied the NFATc1, c‐FOS, CTSK and Nrf2 antibodies (Cambridge, UK). The Keap1 and HO‐1 primary antibodies were provided by Novusbio. P65 and NFATc1 fluorescent antibodies, and the DAPI were obtained from Santa Cruz Biotechnology (Santa Cruz, CA, USA).

### 2.2 Cell extraction

After the cervical dislocation technique of execution, C57BL6/J mice were sedated, and the femoral and tibial muscle tissue was removed. After cutting the epiphysis, the myeloid chamber was flushed with a 1 mL syringe of buffer. The cell suspensions were centrifuged (1500 rpm for 4 min) and resuspended in full α‐MEM media containing M‐CSF at 25 ng/mL in a solution of 1% penicillin–streptomycin and 10% FBS before seeding onto T‐75 flasks for culture. Cells were incubated in a 37°C, 5% CO_2_ incubator to adhere to the wall, and the BMMs were left for 5 days, changing the full α‐MEM media every 2 days.

### 2.3 CCK‐8 assay

The CCK‐8 assay was performed exactly as directed by the manufacturer. 6 × 10^3^ BMMs cells were cultured in full α‐MEM medium (containing 1% penicillin–streptomycin solution, 25 ng/mL M‐CSF, and 10% FBS) for 96 h at various TDH concentrations (0, 5, 10, 15, 20, 25, 30 and 35 μM). Each well received 10 μL of CCK8 reagent and was incubated in the dark for 2 h. An enzyme marker was used to detect the absorbance values (Bethel Holder Technology GmbH, Germany).

### 2.4 Flow cytometry

BMMs were incubated in a culture medium containing TDH (15 and 30 μM) and RANKL (50 ng/mL) for 2 days. Then, the cells are gathered for testing apoptosis according to the manufacturer's guidelines. The apoptotic rate was measured by using the Becton Dickinson FACSAriaII (Becton, Dickinson and Company, USA) flow cytometer.

### 2.5 TRAcP staining and osteoclast differentiation

In a 5% CO_2_ and 95% air environment, 6 × 10^3^ BMMs cells were seeded in 96‐well plates and incubated overnight in a 37°C incubator. The medium was changed every 2 days to achieve BMM fusion. Cells were grown in full α‐MEM with RANKL (50 ng/mL) and stimulated with TDH (0, 5, 10, 15, 20, 25 and 30 μM). To observe TRAcP‐positive multinucleated cells, the cells were immersed in a solution of 4% paraformaldehyde (PFA) for 10 min before being stained with the TRAcP (Sigma–Aldrich, Merck KGaA, Darmstadt, Germany) reagent for 15 min. Cytation5 (BioTek Instrument Incorporation, Winooski, VT, USA) was used to obtain the stained photographs. The cells were then counted and designated as osteoclasts if they had three or more nuclei.

### 2.6 F‐actin staining

6 × 10^3^ BMMs were seeded into 96‐well plates in an osteoclast differentiation medium as above. After 5 days, the cells were fixed. Cells were then permeabilized for 5 min with Triton‐100 before being sealed for 1 h with 3% BSA and stained for 2 h with rhodamine and 5 min with DAPI. Cytation5 was used to image the cells, and analysed by the Image J software.

### 2.7 Acridine orange staining

6 × 10^3^ BMMs were incubated in 96‐well plates for 48 h with TDH (0, 15 and 30 μM), after that, they were further incubated for 30 min with 5 g/mL acridine orange (Sigma) at 37°C in an incubator. Cytation5 microplate readers were employed to analyse the cells.

### 2.8 Bone resorption assay

The bone slices were placed in a culture dish and shaken for 48 h in a 4° shaker with 75% alcohol, PBS and α‐MEM, respectively. The bone slices were dispersed in 96‐well plates, cell control wells were set up, and then 6 × 10^3^ BMMs per well were added into the 96‐well plates. RANKL at a dose of 50 ng/mL was used to stimulate the cells until tiny osteoclasts developed. TDH (15 and 30 μM) was applied to cells in full α‐MEM containing 50 ng/mL for 48 h. PBS was used to wash the bone slices, and surface cells were carefully brushed away using a tiny brush. A typical resorption area was captured using an electron microscope, the Regulus 8100 (Hitachi, TYO, Japan). Quantification analysis was then performed using the Image J software. The cell control group was fixed with 4% PFA, and TRAcP activity staining was conducted as stated in the preceding section.

### 2.9 NFATc1 and P65 nuclear translocation

BMMs were seeded onto culture dishes at a cell density of 1.5 × 10^5^, cultured with varied concentrations of TDH (0, 15 and 30 μM) in full media containing M‐CSF until osteoclasts formed. Finally, they were stimulated for 30 min with RANKL (or they were then starved for 3 h with pure α‐MEM and stimulated with TDH for 1 h). BMMs were then treated for 10 min with 4% PFA. Five minutes after permeabilization with 0.1% Triton‐100 and pre‐staining for 30 min with 3% BSA, the cells were incubated with 1:50 of 0.1% BSA NFATc1 or P65 fluorescent antibodies overnight at 4°C, followed by 5 min of DAPI staining. Images of staining were photographed using a Cytation 5 (BioTek Instrument Incorporation, Winooski, VT, USA).

### 2.10 Molecular docking

TDH's two‐dimensional structure was obtained from PubChem (https://pubchem.ncbi.nlm.nih.gov/) and optimized and stored as a three‐dimensional structure in ChemBio3D 14.0.0.117 using MM2 molecular mechanics. On the PDB website (https://www.rcsb.org/), the 3D structure of receptor protein IKKβ was downloaded. Using PyMOL 2.5.2, the small molecule ligands and water molecules were removed from the above structure and saved as the PDB file. After conducting hydrogenation and charge calculations, MGLTools 1.5.6 was used to further modify protein and small molecule structures, which were then stored in the PDBQT format. The coordinates were placed at the grid box's centre, and the other parameters remained at their default values. For molecular docking, the autodock vina 1.1.2 was utilized. PyMOL 2.5.2 was used to visualize docking with the following settings: num_modes = 20, energy_range = 5, grid centre in IKKβ *x* = 25.29, *y* = −2.582, *z* = −85.608.

### 2.11 RNA extraction and analysis

1 × 10^5^ BMMs per well in a six‐well plate were used to perform osteoclastogenesis assays in the presence or absence of TDH. In a solution containing 50 ng/mL RANKL and M‐CSF, culture until the formation of osteoclasts. Next, cells were lysed by adding Trizol reagent (Thermo Fisher Scientific) on ice. Reverse transcription and amplification were performed using the Extracted Total RNA Kit (RevertAid First Strand cDNA Synthesis Kit, Thermo Scientific™). The concentration of total RNA was measured using NanoDrop™ One/O (Thermo Fisher Scientific). Finally, real‐time quantitative PCR (RT‐PCR) was performed on a LightCycler® 96 system (Roche, Basel, Switzerland). Target genes were compared with an internal reference gene, β‐actin. Positive control wells were used for normalization and statistical analysis. Primers used were listed in Table S1.

### 2.12 Intracellular ROS generation assay

Osteoclast precursors were treated for 30 min with α‐MEM containing 10 μL DCFH‐DA (MCE, Shanghai, China) after 48 h in the presence or absence of TDH (15 and 30 μM). Cytation5 (BioTek Instrument Incorporation, Winooski, VT, USA) was used to examine and evaluate the stained cells.

### 2.13 Western Blot analysis

BMMs were added at a cell density of 5 × 10^6^ onto six‐well plates and incubated. BMMs were starved with serum free α‐MEM for 3 h before being stimulated with 30 μM TDH for 1 h the next day. RANKL was then added to stimulate for 0, 5, 10, 20, 30 and 60 min (short‐acting) or for 1, 3 and 5 days (long‐acting). RIPA was used to extract proteins from lysed cells, and the proteins were then transferred to nitrocellulose membranes by SDS‐PAGE electrophoresis on ice. The membrane was blocked for 1 h using 5% skim milk before being treated with specific antibodies and appropriate secondary antibodies overnight at 4°C. An Image Quant LAS‐4000 system was used to observe target bands (GE Healthcare, Chicago, IL, USA). Image J software was used for photo analysis.

### 2.14 Model of lipopolysaccharide‐induced osteolysis in mice

The groups were as follows: sham (100 mL of PBS administered daily), LPS (LPS 5 mg/kg alternating with 100 mL of PBS), and TDH low‐concentration (LPS 5 mg/kg alternating with 15 mg/kg of TDH). TDH high‐concentration group (LPS 5 mg/kg alternated with 30 mg/kg of TDH). Each set of five C57BL6/J male mice (10 weeks of age) received a daily subcutaneous injection of 100 μL along the herringbone suture of the skull. When the experiment reached day 8, mice were killed through cervical dislocation, and the skull was harvested. Guangxi Medical University's Animal Protection and Welfare Committee accepted the protocol (permit number: 202203005). These animal experiments were carried out in line with the Laboratory Animal Care and Use Guide.

### 2.15 Micro‐CT scan and analysis of mouse skull

The muscular tissue was excised with a knife after the mouse skull was initially fixed with 4% PFA. A Micro‐CT scan was used to scan the mouse's whole skull (SkyScan1176, Bruker). The scanning parameters were as follows: SkyScan NRecon is used with a 50 kV source voltage, a 500 μA source current, a 9 μm pixel size, a 0.5 mm AI filter, and a rotating step of 180 degrees. The 3D pictures were reconstructed with the SkyScan NRecon platform and evaluated with the SkyScan CTAn software (Bruker). For subsequent quantitative and qualitative research, a square region of interest (ROI) surrounding the sagittal suture of the skull was determined. The bone volume to tissue volume ratio (BV/TV) and bone resorption area ratio were calculated.

### 2.16 Histological analysis

For 3 weeks, mouse skulls were fixed and decalcified in 14% EDTA before being paraffin‐embedded. To stain with H&E, TRAcP, and immunohistochemical labelling, all tissues were fixed in paraffin and sliced into about 4–5 μm. KFPRO scanner (KONFONG Biotechnology International Ltd., Ningbo, China) was used to capture images, which were then visualized using K‐Viewer software (Konfong Biotechnology International Ltd.).

### 2.17 Statistical analysis

For statistical and graphing quantitative analysis, GraphPad Software (San Diego, CA, USA) was utilized. One‐way ANOVA plus Tukey's test or Kruskal–Wallis analysis (nonparametric ANOVA) plus Dunn's multiple comparisons were employed to assess differences between more than two groups. A two‐way ANOVA was used to assess the effects of time and treatment groups, with a *p* < 0.05 considered statistically significant.

## 3 RESULTS

### 3.1 TDH inhibits RANKL‐induced OC differentiation in vitro

**FIGURE 1:**
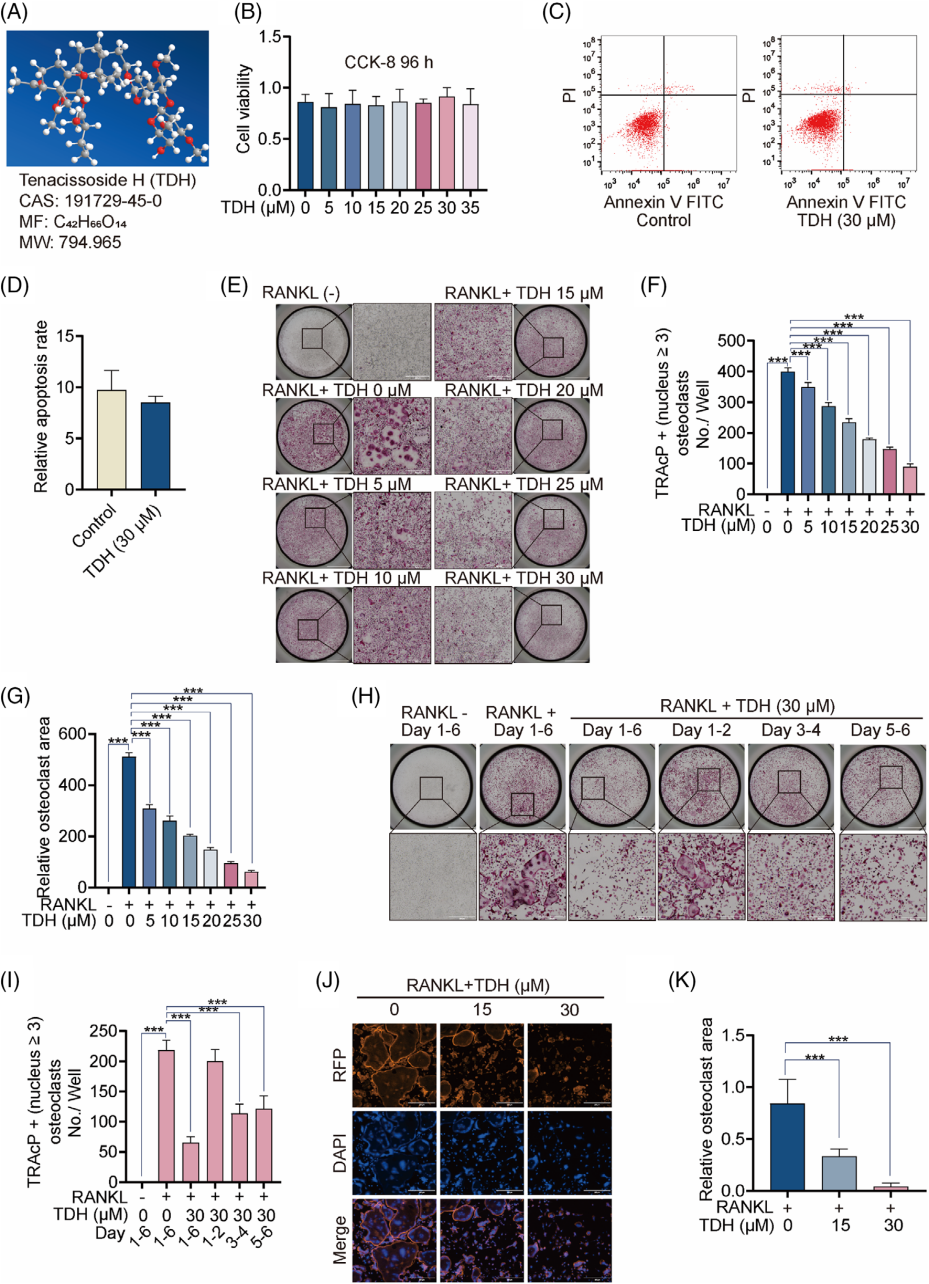
TDH inhibits RANKL‐induced OC differentiation in vitro. (A) TDH molecular structure in 3D. (B) CCK8 detected the effect of TDH on the viability of BMMs. (C) Apoptosis in OCs was measured by flow cytometry. (D) Quantification of cell apoptosis. (E) After 6 days of culture, typical photos of TRAcP staining. Note that BMMs were co‐stimulated with varied concentrations of TDH with M‐CSF (25 ng/mL) and RANKL (50 ng/mL). (F, G) TRAcP‐positive OCs were analysed quantitatively in number and in area (*n* = 3). (H) Images of typical OCs at various stages of development. (I) TRAcP‐positive OCs were analysed quantitatively (*n* = 3). (J) Representative photos of rhodamine‐phalloidin staining of the podosome belts in OCs. (K) The area of all osteoclasts was measured (*n* = 3). All statistical histograms are expressed as means and standard deviations. *p < 0.05, **p < 0.01, ***p < 0.001. RANKL, receptor activator of the nuclear factor‐κB ligand; TDH, tenacissoside H; TRAcP, tartrate‐resistant acid phosphatase.

Three‐dimensional (3D) TDH molecular structure was presented in Figure 1A. TDH had no detrimental effect on BMM survival in the CCK8 experiment (Figure 1B). TDH was shown to have no negative effect on the apoptosis of BMMs (Figure 1C,D). The TRAcP assay revealed that TDH reduced OC differentiation dose‐dependently (Figure 1E–G). TDH, in particular, prevented the formation of OCs at the mid‐stages and late‐stages but had no effect at the early‐stages (Figure 1H,I). These findings suggest that TDH reduces osteoclast development in the middle and late stages. By RANKL stimulation, mature OCs formed obvious podosome belts, and the podosome belt area of OCs was considerably reduced after the addition of 15 μM and 30 μM TDH (Figure 1J,K).

Furthermore, the results of alkaline phosphatase staining and alizarin red staining (Figure S1A–D) showed that TDH also had no significant effects on the formation of osteoblastic mineralized nodules.

### 3.2 TDH inhibits intracellular acid levels and bone resorption

**FIGURE 2:**
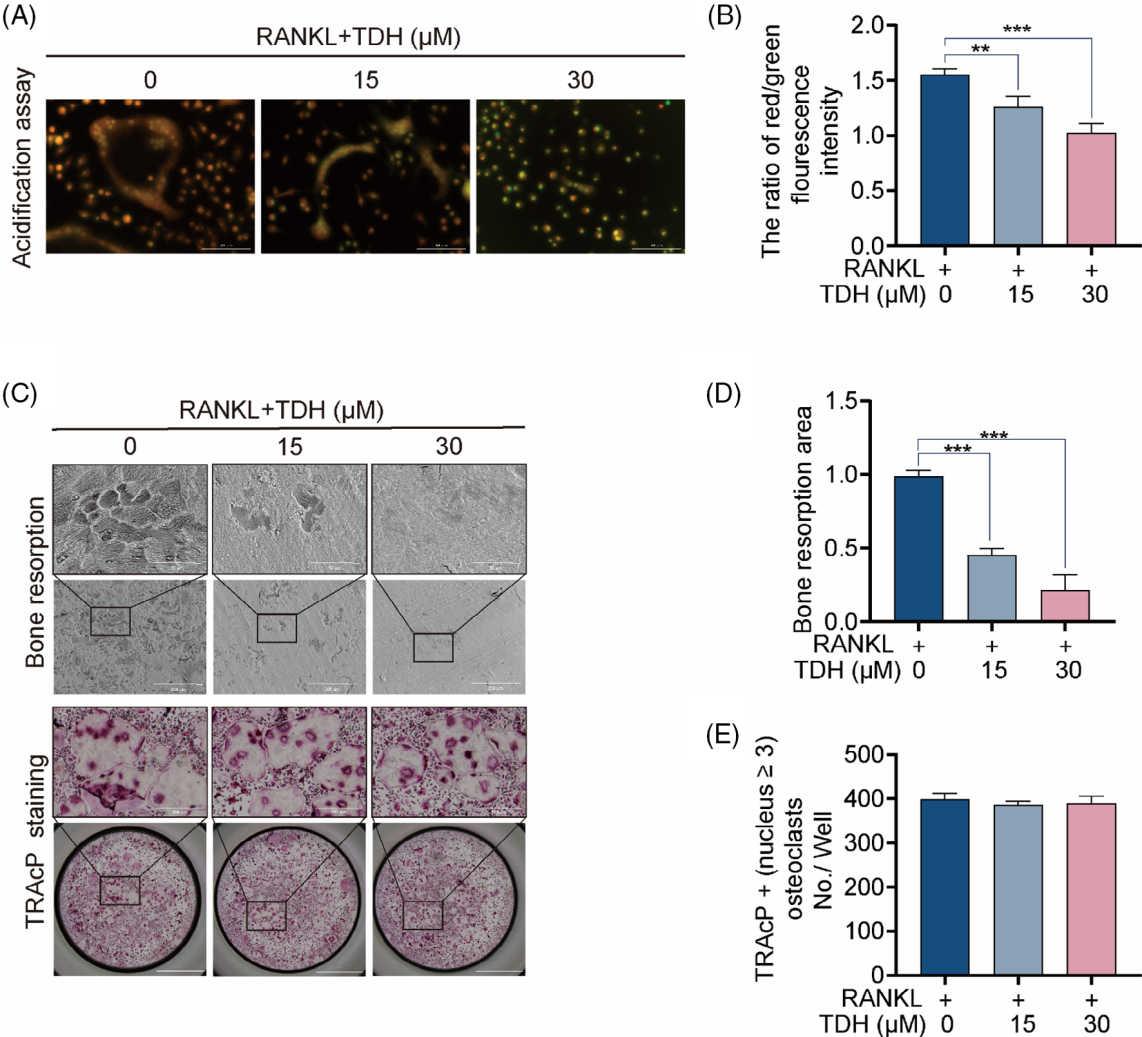
TDH inhibits intracellular acid levels and bone resorption. (A) Representative images of acids secretion. (B) Fluorescence intensity ratio of red to green. (C) Bone resorption pits and TRAcP staining on bovine bone slices were shown in representative micrographs. (D) Bone resorption pit area assays (*n* = 3). (E) TRAcP‐positive OCs were analysed quantitatively (*n* = 3). All statistical histograms were expressed as means and standard deviations. *p < 0.05, **p < 0.01, ***p < 0.001. RANKL, receptor activator of the nuclear factor‐κB ligand; TDH, tenacissoside H; TRAcP, tartrate‐resistant acid phosphatase.

Next, we found that TDH significantly reduced the intensity of intracellular acidic fluorescence (Figure 2A,B). In addition, TDH intervention significantly reduced the area of osteoclastic bone resorption in bovine bone slices when compared to the RANKL‐stimulated group (Figure 2C–E).

### 3.3 TDH inhibits the expression of OC‐related genes and proteins in vitro

**FIGURE 3:**
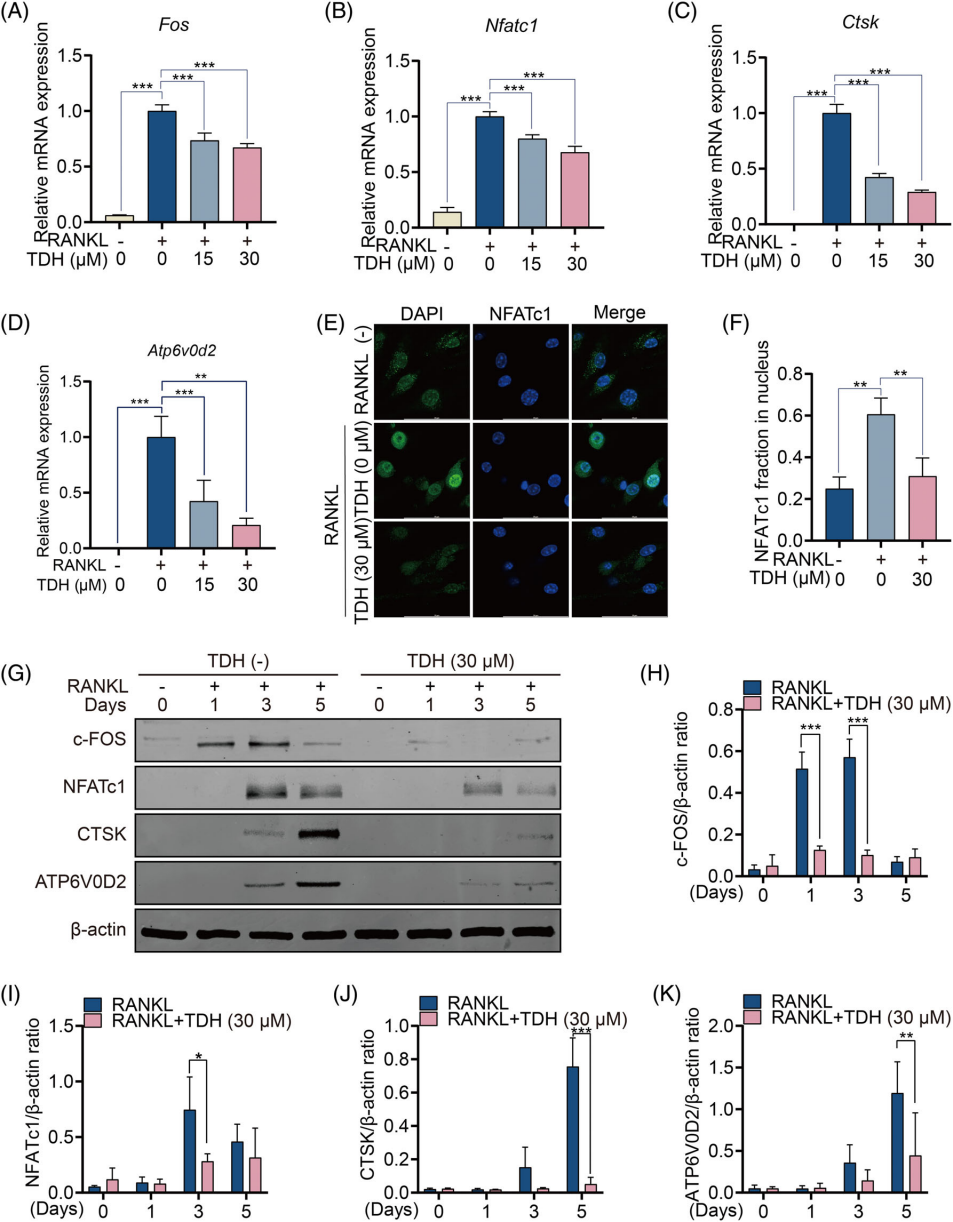
TDH inhibits the expression of OC‐related genes and proteins in vitro. (A–D) The gene expressions of *Fos*, *Nfatc1*, *Ctsk* and *Atp6v0d2* in osteoclastogenesis were assessed by RT‐qPCR (*n* = 3). (E) Images of NFATc1 nuclear translocations induced by RANKL exposed or not to TDH. (F) Measurement of the relative fraction of NFATc1 in nuclei. (G) The protein expressions of c‐FOS, NFATc1, CTSK and ATP6V0D2 were assessed by Western blot. (H–K) The ratios of c‐FOS, NFATc1, CTSK and ATP6V0D2 relative to β‐actin, respectively (*n* = 3). All statistical histograms were expressed as means and standard deviations. *p < 0.05, **p < 0.01, ***p < 0.001. ATP6V0D2, ATPase H+ transporting V0 subunit D2; c‐FOS, proto‐oncogene c‐Fos; CTSK, cathepsin K; NFATc1, nuclear factor of activated T cells 1; RANKL, receptor activator of the nuclear factor‐κB ligand; TDH, Tenacissoside H.

The transcription of RANKL‐related genes was studied, and the findings revealed that a number of OC‐specific genes, including *Fos*, *Nfatc1*, *Ctsk* and *Atp6vod2*, were considerably down‐regulated by TDH (Figure 3A–D). In addition, TDH was found to suppress RANKL‐induced nuclear translocation of NFATc1 (Figure 3E,F). Further, the protein expression of c‐FOS, NFATc1, CTSK and ATP6V0D2 was also down‐regulated by TDH (Figure 3G–K).

### 3.4 TDH suppresses RANKL‐induced NF‐κB signalling pathway in vitro

Next, the role of TDH in the MAPK and NF‐κB pathways immediately after RANKL activation was investigated. TDH had no influence on the phosphorylation of P38, ERK or JNK at any time, as demonstrated by Western blot analysis (Figure S2A–D).

**FIGURE 4:**
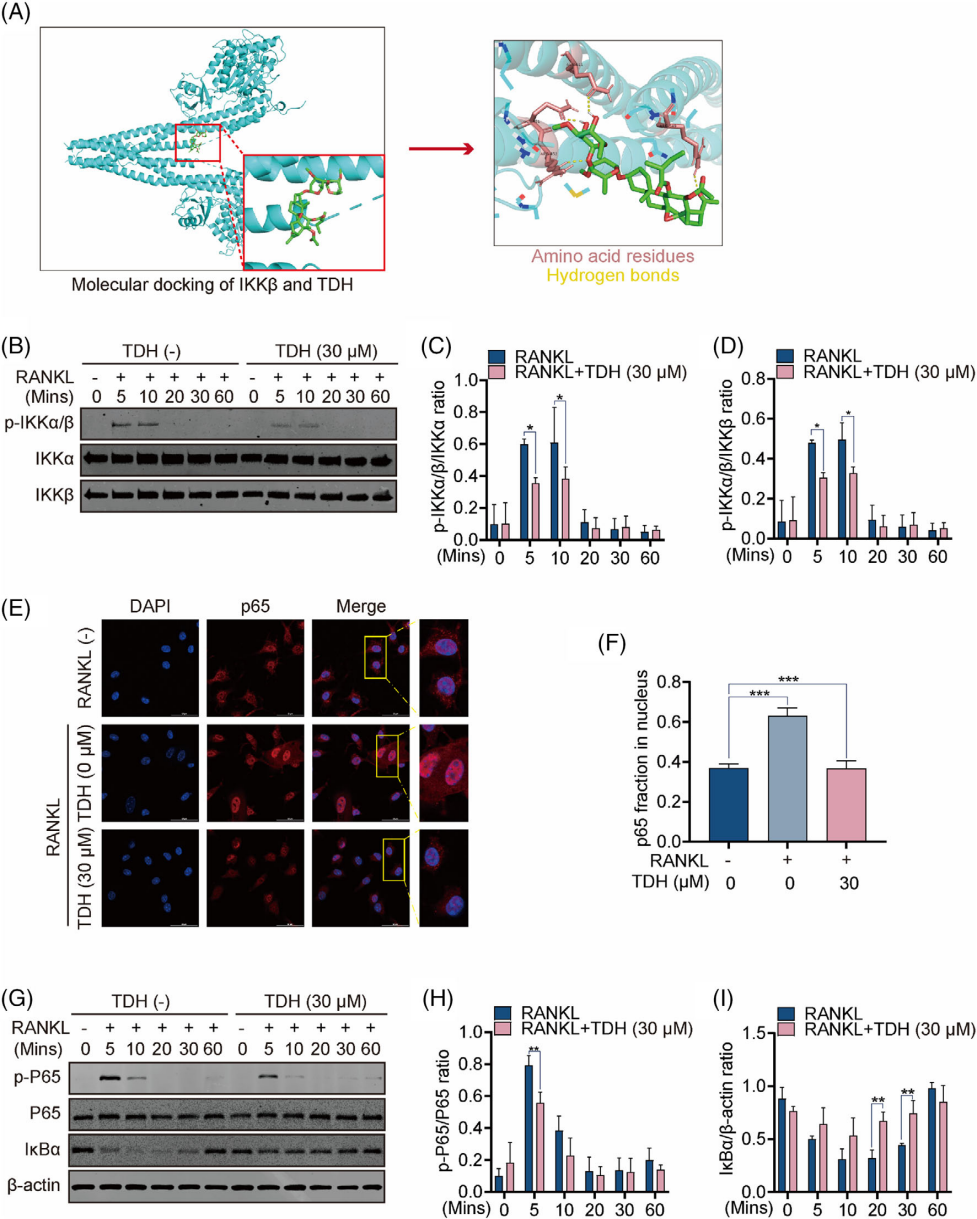
TDH suppresses RANKL‐induced NF‐κB signalling pathway in vitro. (A) Molecular docking experiments showed TDH's probable interaction with IKKβ. (B) The protein expressions of p‐IKKα/β, IKKα, IKKβ in osteoclastogenesis were assessed by Western blot. (C, D) The ratios of p‐IKKα/β/IKKα, p‐IKKα/β/IKKβ (*n* = 3). (E) Images of RANKL‐induced P65 nuclear translocations in the absence and presence of TDH. (F) Measurement of the relative fraction of P65 in the nucleus. (G) The protein expressions of p‐P65, P65, IκBα and β‐actin were assessed by Western blot. (H, I) The ratios of p‐P65/P65 and IκBα/β‐actin (*n* = 3). All statistical histograms were expressed as means and standard deviations. *p < 0.05, **p < 0.01, ***p < 0.001. IKK, inhibitor of kappa B kinase; NF‐κB, nuclear factor‐κB; RANKL, receptor activator of the nuclear factor‐κB ligand; TDH, Tenacissoside H.

We continued to investigate whether TDH impacts NF‐κB upstream target proteins. First, we used molecular docking experiments to predict TDH's probable interaction with IKKβ, which revealed the formation of five hydrogen bonds between TDH and IKKβ amino acid residues GLN‐451, GLN‐611, ARG‐452 and ARG‐549. The binding energies were − 6.1 kcal mol^−1^, indicative of a high binding activity (Figure 4A). Western blot analysis demonstrated that TDH had a considerable impact on IKKα/β phosphorylation, 5 min after RANKL stimulation (Figure 4B–D). TDH was also discovered to suppress RANKL‐induced nuclear translocation of P65 (Figure 4E,F). In addition, Western blot analysis showed that TDH had a noticeable impact on the phosphorylation of P65 after 5 min, but its effect on the degradation of IκBα did not occur until 20 min (Figure 4G–I).

### 3.5 TDH inhibits LPS‐induced osteoclast differentiation in vitro

**FIGURE 5:**
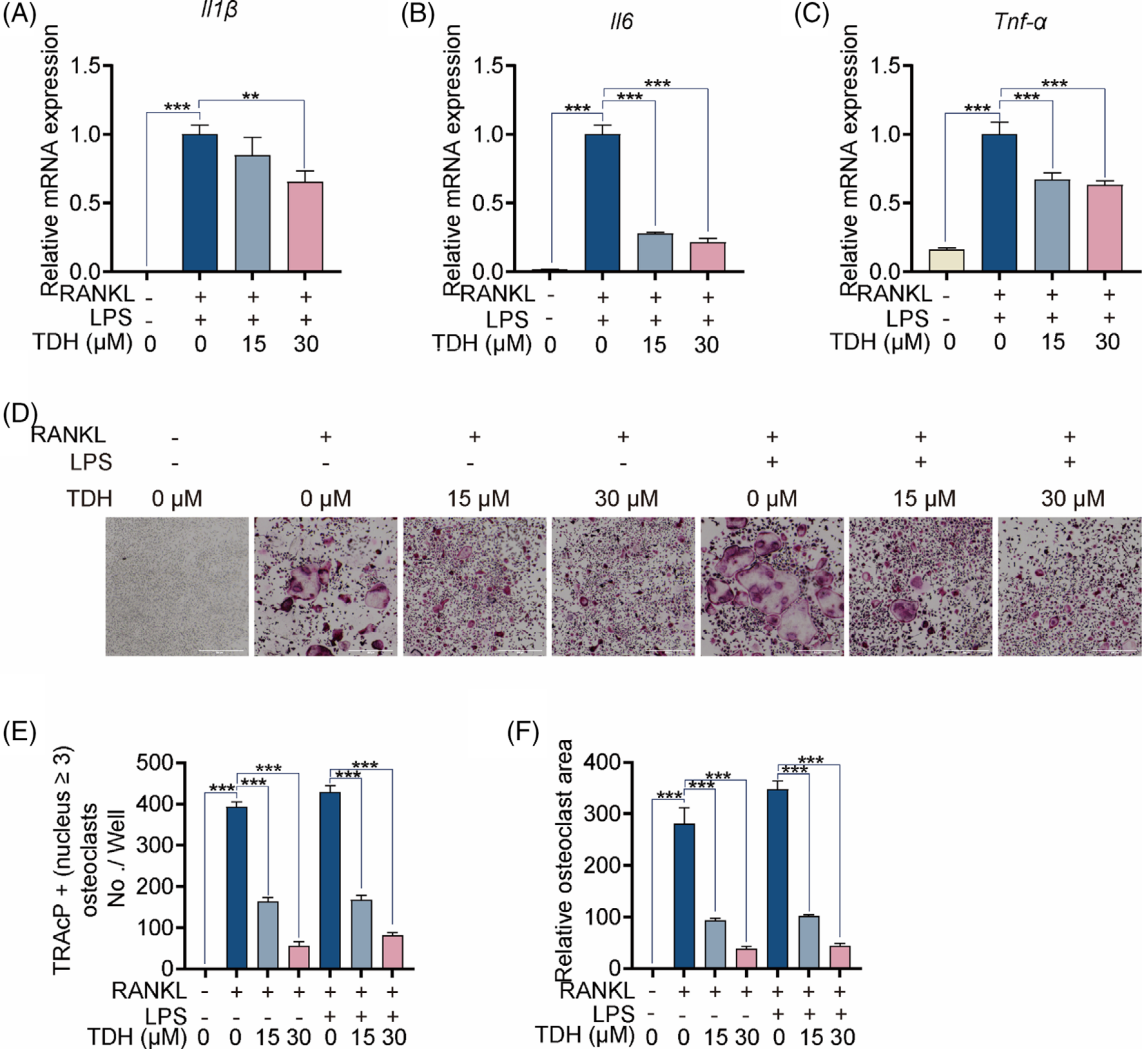
TDH inhibits LPS‐induced osteoclast differentiation in vitro. (A–C) The gene expressions of *Il1β*, *Il6* and *Tnf‐α* in osteoclastogenesis were assessed by RT‐qPCR (*n* = 3). (D) Typical TRAcP staining images with and without LPS. (E, F) TRAcP‐positive OCs were subjected to quantitative analyses in number and area (*n* = 3). All statistical histograms were expressed as means and standard deviations. *p < 0.05, **p < 0.01, ***p < 0.001. IL‐1β, interleukin 1β; IL‐6, interleukin 6; RANKL, receptor activator of the nuclear factor‐κB ligand; TDH, Tenacissoside H; TRAcP, tartrate‐resistant acid phosphatase; TNF‐α, tumour necrosis factor α.

LPS‐induced inflammatory cytokines were released during OC development.^23^ Consistently, we found that TDH inhibited inflammatory factor secretion, including IL‐1β, IL‐6 and TNF‐α (Figure 5A–C). Additionally, when pre‐OCs were first stimulated with RANKL for 2 days before being stimulated with LPS for 24 h, the degree of OC differentiation increased dramatically when compared to the control group, whereas TDH was found to inhibit this effect (Figure 5D–F).

### 3.6 TDH inhibits LPS‐induced ROS levels in vitro

**FIGURE 6:**
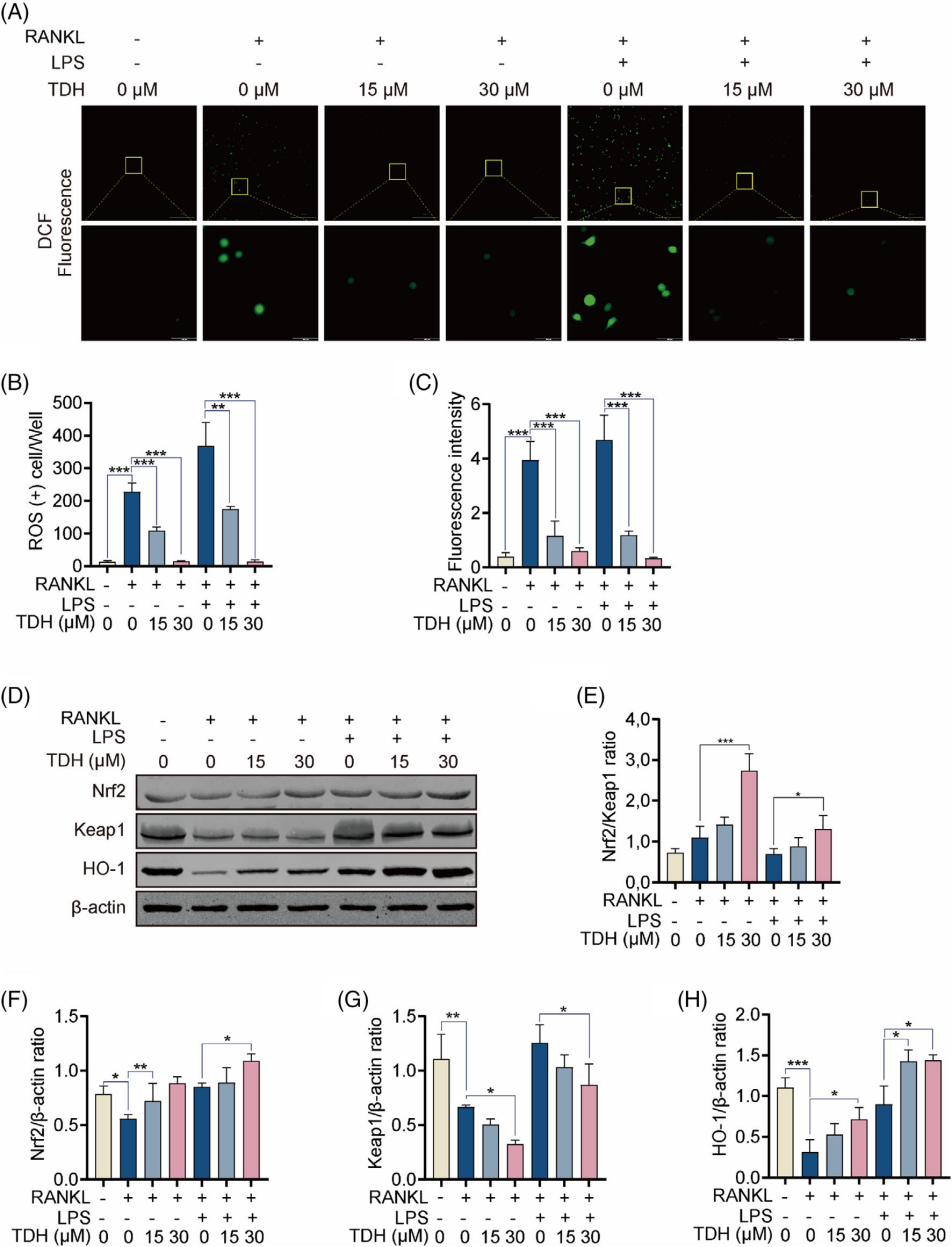
TDH inhibits LPS‐induced ROS levels in vitro. (A) DCF was used to measure intracellular ROS levels. (B, C) DCF fluorescence intensities on cells per well were quantified. (D) The protein expressions of Nrf2, Keap1 and Hmox1 were assessed by Western blot. (E–H) The ratios of Nrf2/Keap1 and Nrf2, Keap1 and Hmox1 to β‐actin (*n* = 3). All statistical histograms were expressed as means and standard deviations. *p < 0.05, **p < 0.01, ***p < 0.001. DCF, 2′,7′‐dichlorofluorescein; HO‐1, heme oxygenase 1; Keap1, Kelch‐like ECH‐associated protein; Nrf2, nuclear factor erythroid‐derived 2‐like 2; RANKL, receptor activator of the nuclear factor‐κB ligand; ROS, reactive oxygen species; TDH, Tenacissoside H.

Pre‐OCs were first stimulated with RANKL for 2 days before being stimulated with LPS for 24 h. TDH (15 and 30 μM) was found to reduce DCF fluorescence intensity, as a measurement of ROS levels induced by LPS (Figure 6A–C). Western blot analyses revealed that protein expression of Nrf2/keap1, Nrf2 and HO‐1 increased as TDH concentration increased, whereas Keap1 protein expression decreased steadily (Figure 6E–H). These findings imply that TDH can inhibit ROS production during LPS‐induced osteoclastic differentiation.

### 3.7 TDH reverses LPS‐induced bone loss in mice calvaria in vivo

**FIGURE 7:**
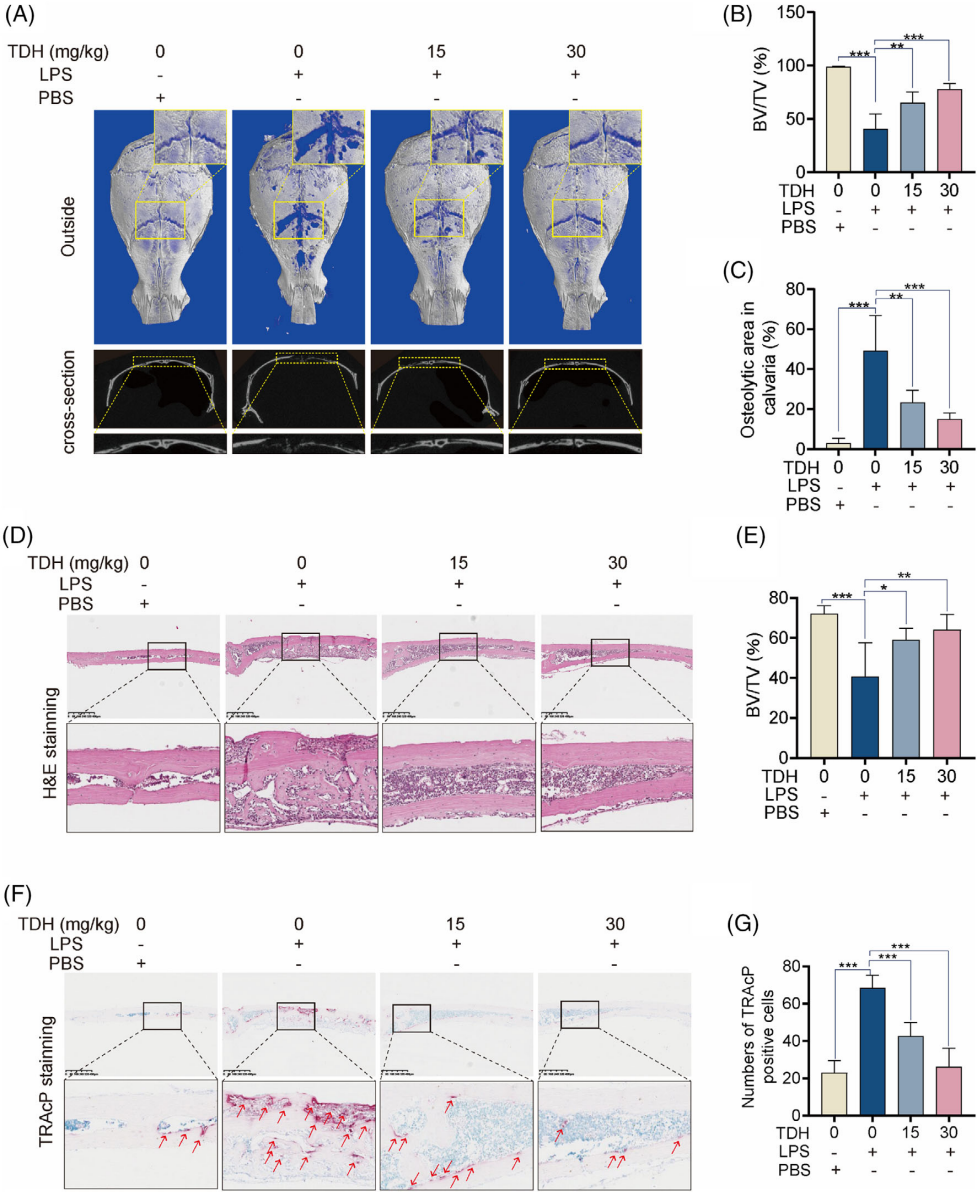
TDH reverses LPS‐induced bone loss in mice calvaria in vivo. (A) Micro‐CT scan 3D structure and cross‐sectional pictures. (B, C) The BV/TV volume and osteolytic area were quantified (*n* = 5). (D) Representative H&E‐staining pictures of mouse calvarial tissue sections. (E) Quantification of BV/TV volume in H&E‐stained sections (*n* = 3). (F) Representative TRAcP staining photographs of mouse calvarial tissue slices. (G) OCs quantification in TRAcP‐stained sections (*n* = 3). All statistical histograms were expressed as means and standard deviations. *p < 0.05, **p < 0.01, ***p < 0.001. BV, bone volume; BV/TV, bone volume fraction; LPS, lipopolysaccharide; PBS, phosphate buffer saline; TDH, tenacissoside H; TV, total volume.

In vivo study showed that TDH at 15 mg/kg and 30 mg/kg both reduced LPS‐induced calvarial bone loss in mice to varying degrees, according to 3D structure and cross‐sectional pictures (Figure 7A). TDH at 30 mg/kg was more effective at lowering BV/TV and osteolysis area (Figure 7B,C). Histological analysis was then performed. Sections stained with H&E indicated that the surface of the damaged bone was much greater in the LPS‐injected group compared to the sham group, and that TDH was found to reduce the levels of bone erosion (Figure 7D,E). Compared to the LPS group, TDH also reduced the total number of osteoclasts per field of view (Figure 7F,G).

**FIGURE 8:**
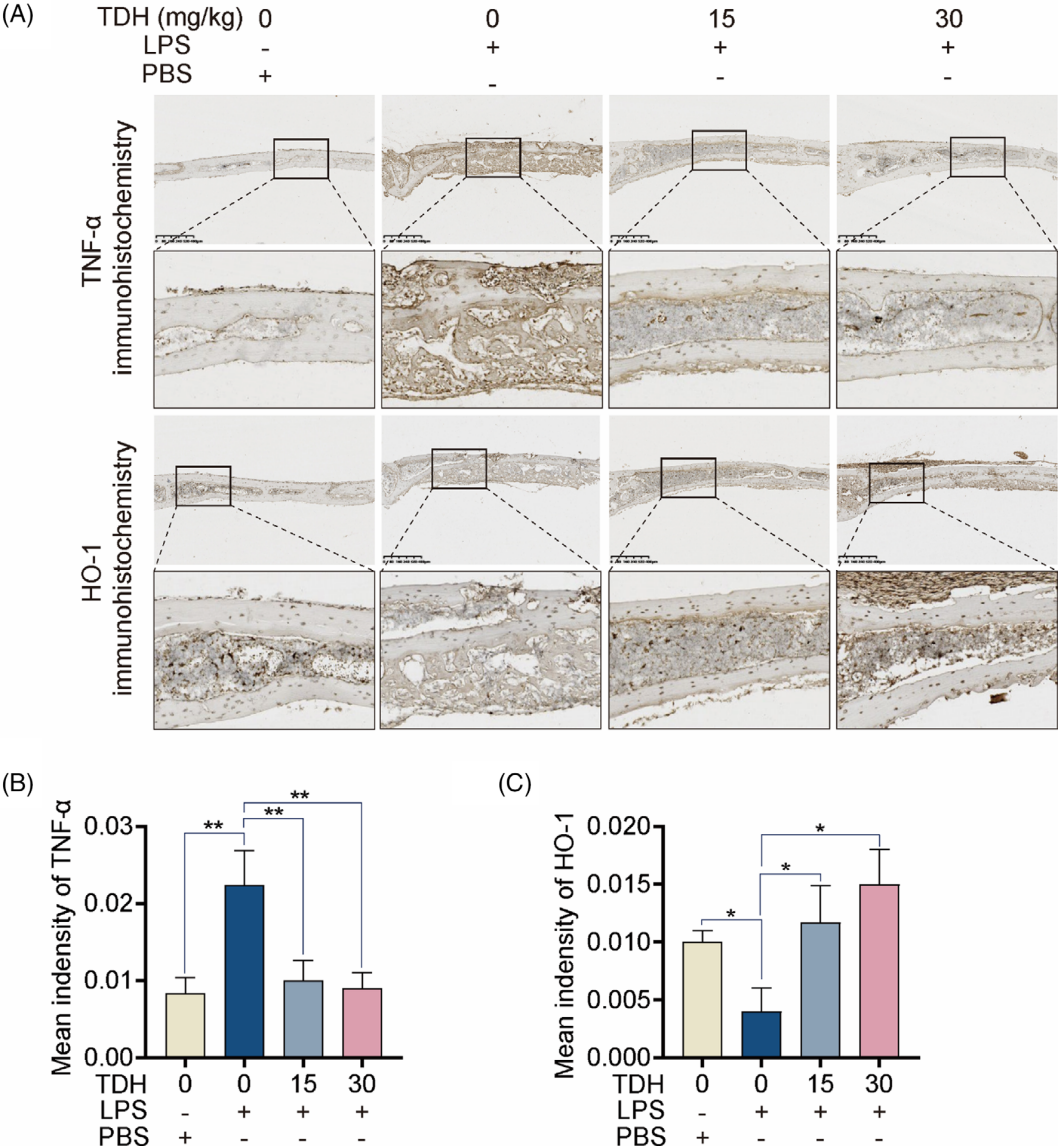
TDH decreases TNF‐α and increasing HO‐1 expression in mouse calvaria in vivo. (A) Immunohistochemical images of TNF‐α and HO‐1 in calvaria of LPS‐induced cranial osteolysis in mice. (B, C) Quantitative mean optical density of TNF‐α and HO‐1 protein expression (*n* = 3). All statistical histograms were expressed as means and standard deviations. *p < 0.05, **p < 0.01, ***p < 0.001. HO‐1, heme oxygenase 1; LPS, lipopolysaccharide; PBS, phosphate buffer saline; TDH, tenacissoside H; TNF‐α, tumour necrosis factor α.

We also performed immunohistochemistry staining of TNF‐α and HO‐1 on mouse skull sections. TDH was found to decrease TNF‐α protein expression and increase HO‐1 protein expression in vivo (Figure 8A–C). Collectively, TDH appears to have anti‐inflammatory and antioxidant effects, thus reducing LPS‐mediated inflammatory bone loss in vivo.

## 4 DISCUSSION

Inflammatory osteolysis is a common orthopaedic condition, and its treatment and prevention have long been a focus of research.^24^ It is believed that inflammatory osteolysis is influenced by cytokines released in the inflammatory microenvironment, leading to increased OC recruitment and activation.^25^ Thus, one of the most significant approaches in the treatment of localized osteolysis has been on the inhibition of inflammatory cytokines on OC development and activity.^26^, ^27^

OCs attach to the bone matrix after emerging from the monocyte/macrophage haematopoietic spectrum, destroying hydroxyapatite and collagen in bone tissue by secreting degrading enzymes such as V‐ATPases and cathepsin K.^28^, ^29^ Previous research has shown that TDH has anti‐inflammatory and anti‐tumour properties.^30^ In this study, TDH was shown to have no negative effect on the apoptosis and survival of BMMs. We investigated the effect of TDH on osteoclast formation and bone resorption. TDH was found to dramatically reduce the numbers of TRAcP‐positive cells and the sizes of podosome belts. TDH also reduced bone resorption and intracellular acidic levels.

The NF‐κB pathway is significant for osteoclast development.^31^ The IKK complex is required for the activation of the NF‐κB signalling pathway, via phosphorylating IKKα, IKKβ and a regulatory subunit NEMO.^32^ This process, in turn, causes IκB proteins to be rapidly ubiquitinated and degraded via the ubiquitin‐mediated proteasomal degradation pathway. Upon the degradation of IκB, NF‐κB proteins can move from the cytoplasm to the nucleus and bind to their respective DNA binding sites, where they regulate the transcription of a diverse collection of downstream target genes.^33^, ^34^ It has been shown that TDH is beneficial in treating a diverse range of disorders via mediating NF‐κB signalling pathway in various cell types.^22^, ^35^ We postulated that TDH might block IKK activation and thereby reduce the NF‐κB signalling pathway. A molecular docking experiment confirmed the interaction of TDH with IKK. Consistently, the Western blot assays demonstrated that TDH had an impact on the phosphorylation of IKKα/β and P65, as well as P65 nuclear translocation.

NFATc1, is a transcribed factor that controls gene expression and promotes the synthesis and function of OCs.^36^ Interestingly, TDH was found to reduce NFATc1 expression, NFATc1 nuclear translocation and NFATc1 activation. Consistently, the expressions of OCs‐related genes and proteins such as c‐FOS, CTSK, and ATP6V0D2 were decreased in vitro.

LPS has been linked to several types of bone loss caused by inflammation.^37^ LPS promotes the formation of OCs and accelerates bone resorption,^38^ accompanied by the release of inflammatory factors.^39^ The number of OCs increased when LPS was applied to RANKL‐treated OCs, indicating that LPS has an additive effect on RANKL‐mediated OC differentiation and is ineffective alone in OC progenitor cells.^40^, ^41^ Interestingly, we have found that TDH was able to reduce the release of LPS‐mediated inflammatory factors and the rate of osteoclast differentiation mediated by RANKL and LPS.

Several studies have shown that ROS are associated with bone metabolism. Their overproduction causes oxidative stress, which accelerates OC differentiation and activation, resulting in bone loss.^42^, ^43^ Nrf2 (nuclear factor‐erythroid 2 related factor 2) is necessary for host cell oxidative stress resistance and bone homeostasis. When cells are stimulated, Nrf2 is freed from its inhibitor Keap1 and translocates to the nucleus, where it binds to antioxidant response elements that promote the synthesis of antioxidant enzymes such as heme oxygenase‐1 (HO‐1) and others, which physiologically prevent osteoclast overgrowth by down‐regulating ROS and thus generating negative feedback signals on osteoclast formation.^44^, ^45^, ^46^ We found that TDH was able to lower the ROS levels with and without LPS in a concentration‐dependent manner. Consistently, the protein expressions of Nrf2/Keap1, Nrf2 and HO‐1 were increased, whereas the protein levels of Keap1 decreased as TDH concentration increased. These findings imply that TDH can reduce ROS accumulation during LPS‐induced osteoclastic differentiation.

We employed a mouse model of osteolysis with LPS injection to investigate the therapeutic effect of TDH in vivo. Micro‐CT and histology results indicated that TDH considerably reduced bone surface erosion compared to the LPS‐injected group. Consistently, TDH‐treated group had a lower overall number of osteoclasts than the LPS‐injected group. TDH also boosted HO‐1 protein expression while inhibiting TNF‐α protein expression in vivo, as evidenced by immunohistochemistry staining of mouse skull slices, which is consistent with in vitro results.

**FIGURE 9:**
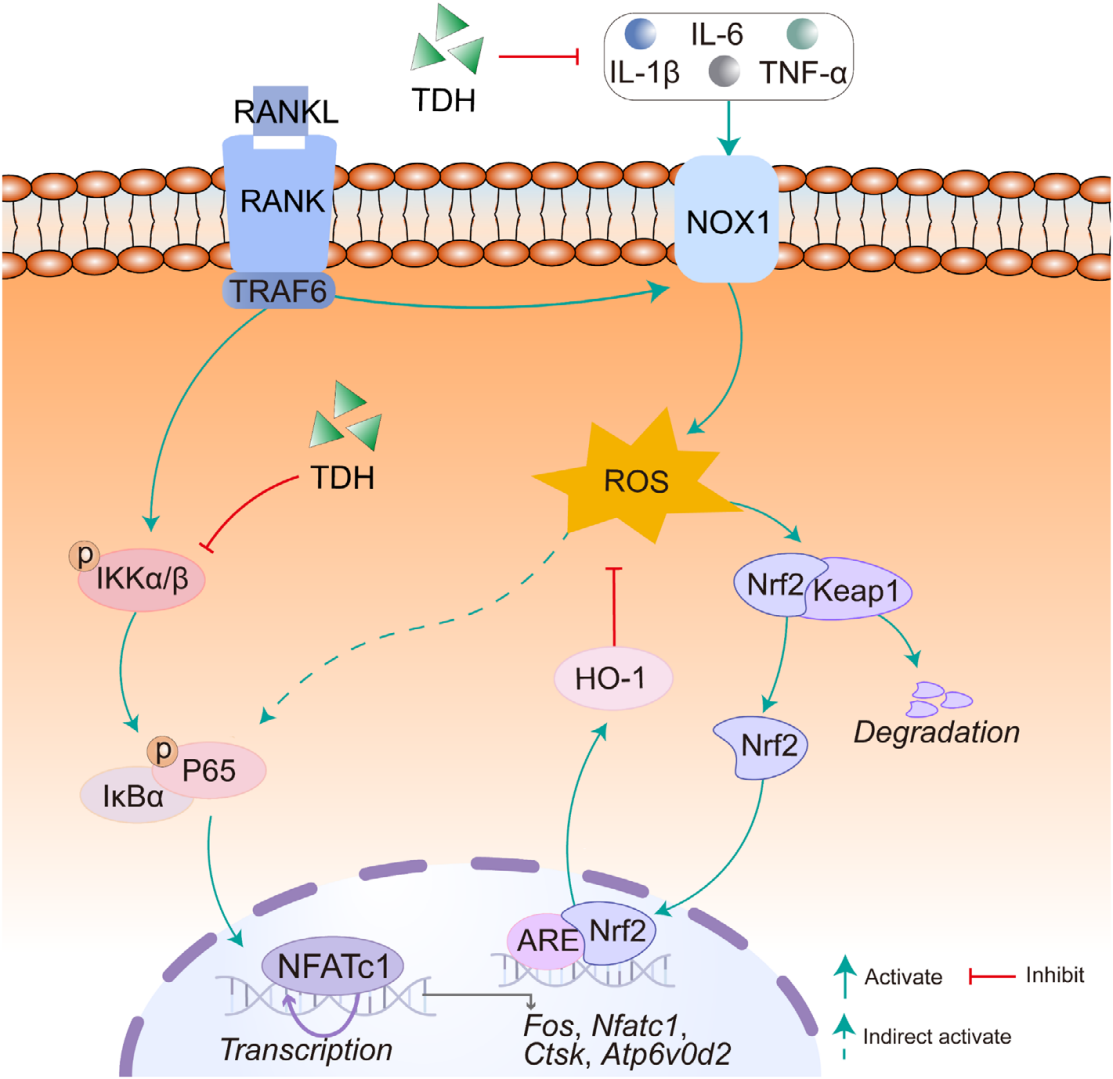
The proposed mechanism of TDH on the suppression of osteoclastogenesis is shown. ATP6V0D2, ATPase H+ transporting V0 subunit D2; CTSK, cathepsin K; c‐FOS, proto‐oncogene c‐Fos; Keap1, Kelch‐like ECH‐associated protein; Nrf2, nuclear factor erythroid‐derived 2‐like 2; NFATc1, nuclear factor of activated T cells 1; HO‐1, heme oxygenase 1; IL‐1β, interleukin 1β; IL‐6, interleukin 6; RANKL, receptor activator of the nuclear factor‐κB ligand; TDH, tenacissoside H; TNF‐α, tumour necrosis factor α.

In summary, our findings showed that TDH inhibits osteoclastogenesis and bone resorption capacity in vitro via its inhibitory effects on NF‐κB pathway, inflammatory factor secretion, intracellular ROS formation, NFATc1 expression and its downstream genes in osteoclasts (Figure 9). In addition, TDH inhibited LPS‐induced inflammatory osteolysis in vivo. Thus, TDH could represent a potential drug candidate for inflammatory osteolytic disorders.

## AUTHOR CONTRIBUTIONS

**Xiaoxiao Xie**: writing‐first draft, visualization. **Weiwei Chen**: data curation. **Minglian Xu**: methodology. **Junchun Chen**: investigation. **Tao Yang**: formal analysis. **Chaofeng Wang**: carried out the experiment. **Yuangang Su**: software. **Jinmin Zhao**: project administration, funding acquisition. **Jiake Xu**: writing‐review & editing. **Qian Liu**: supervision, writing‐review & editing, funding acquisition. Thanks to biorender.com for the graphical abstract image material.

## CONFLICT OF INTEREST STATEMENT

This paper is the outcome of independent research conducted under the supervision of my tutor. Except for those cited in the text, this work contains no scientific achievements written or published by other individuals or groups. Individuals and groups that have made significant contributions to the research of this publication have been identified. I am fully aware that I am personally liable for this statement.

## Supporting information


**Table S1.** The primers used in this study.
**Figure S1.** TDH does not affect osteoblasts formation and mineralization. (A) Representative images of ALP staining in the presence of TDH (15 and 30 μM) for 7 days. (B) Quantification intensity of the ALP staining after TDH treatment. (C) Representative images of Alizarin Red S in the presence of TDH (15 and 30 μM) for 21 days. (D) Alizarin Red S quantification after TDH treatment. All statistical histograms were expressed as mean and standard deviation. *p < 0.05, **p < 0.01, ***p < 0.001. TDH: tenacissoside H, ALP: alkaline phosphatase, ARS: alizarin red S.
**Figure S2.** TDH does not suppress MAPK pathway during osteoclastogenesis. (A) The protein expression levels of P38, ERK, JNK and their phosphorylated forms were measured by Western blot. (B–D) The ratio of p‐P38/P38, p‐ERK/ERK, p‐JNK/JNK (*n* = 5). All statistical histograms were expressed as means and standard deviations. *p < 0.05, **p < 0.01, ***p < 0.001. TDH: tenacissoside H, RANKL: receptor activator of the nuclear factor‐κB ligand, MAPK: mitogen‐activated protein kinases.

## Data Availability

The original contributions presented in the study are included in the article/Supplementary Material. Further inquiries can be directed to the corresponding authors.
