# Supplementary material for: IKK/NF‐κB and ROS signal axes are involved in Tenacissoside H mediated inhibitory effects on LPS‐induced inflammatory osteolysis
Source: Cell Prolif. 2023 Aug 8;57(1):e13535. doi: 10.1111/cpr.13535 (PMC10771108; doi:10.1111/cpr.13535)
Supplement: Supplementary file 1 — Table S1. The primers used in this study. Figure S1. TDH does not affect osteoblasts formation and mineralization. (A) Representative images of ALP staining in the presence of TDH (15 and 30 μM) for 7 days. (B) Quantification intensity of the ALP staining after TDH treatment. (C) Representative images of Alizarin Red S in the presence of TDH (15 and 30 μM) for 21 days. (D) Alizarin Red S quantification after TDH treatment. All statistical histograms were expressed as mean and standard deviation. *p < 0.05, **p < 0.01, ***p < 0.001. TDH: tenacissoside H, ALP: alkaline phosphatase, ARS: alizarin red S. Figure S2. TDH does not suppress MAPK pathway during osteoclastogenesis. (A) The protein expression levels of P38, ERK, JNK and their phosphorylated forms were measured by Western blot. (B–D) The ratio of p‐P38/P38, p‐ERK/ERK, p‐JNK/JNK (n = 5). All statistical histograms were expressed as means and standard deviations. *p < 0.05, **p < 0.01, ***p < 0.001. TDH: tenacissoside H, RANKL: receptor activator of the nuclear factor‐κB ligand, MAPK: mitogen‐activated protein kinases. [file CPR-57-e13535-s001.docx]

## Supplementary material

Supplementary Table 1

| The primers used in this study | | |
| --- | --- | --- |
| Primers for qRT-PCR | |  |
| Target Gene | Primer Sequence (5ʹ- 3ʹ) | |
|  | Forward | Reverse |
| *Fos* | CCAGTCAAGAGCATCAGCAA | AAGTAGTGCAGCCCGGAGTA |
| *Nfatc1* | GGTGCTGTCTGGCCATAACT | GAAACGCTGGTACTGGCTTC |
| *Ctsk* | AGGCGGCTATATGACCACTG | TCTTCAGGGCTTTCTCGTTC |
| *Atp6v0d2* | GTCCCATTCTTGAGTTTGAGG | GGATAGAGTTTGCCGAAGGTT |
| *β-actin* | TCCTCCCTGGAGAAGAGCTA | ATCTCCTTCTGCATCCTGTC |
| *Il1β* | GCAACTGTTCCTGAACTCAACT | ATCTTTTGGGGTCCGTCAACT |
| *Il6* | TAGTCCTTCCTACCCCAATTTCC | TTGGTCCTTAGCCACTCCTTC |
| *Tnf-α* | GACGTGGAACTGGCAGAAGAG | TTGGTGGTTTGTGAGTGTGAG |

*Fos*: proto-oncogene c-Fos, *Nfatc1*: nuclear factor of activated T cells 1, *Ctsk*: cathepsin K, *Atp6v0d2*: ATPase H+ transporting V0 subunit D2, *Il1β*: interleukin 1β, *Il6*: interleukin 6, *Tnf-a*: tumor necrosis factor α.


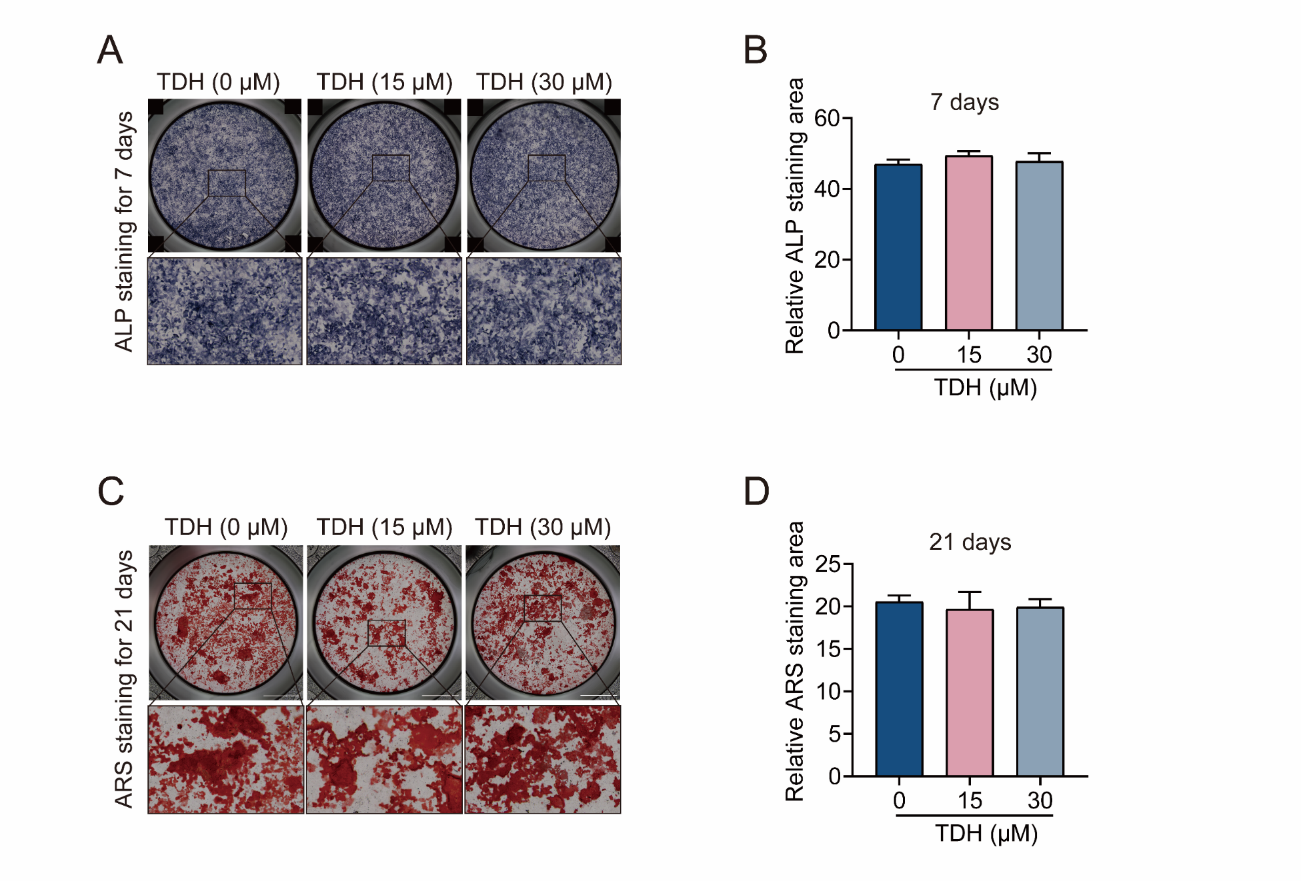


Supplementary Fig 1. TDH does not affect osteoblasts formation and mineralization. (A) Representative images of ALP staining in the presence of TDH (15 and 30 μM) for 7 days. (B) Quantification intensity of the ALP staining after TDH treatment. (C) Representative images of Alizarin Red S in the presence of TDH (15 and 30 μM) for 21 days. (D) Alizarin Red S quantification after TDH treatment. All statistical histograms were expressed as mean and standard deviation. * p < 0.05, ** p < 0.01, *** p < 0.001. TDH: tenacissoside H, ALP: alkaline phosphatase, ARS: alizarin red S.


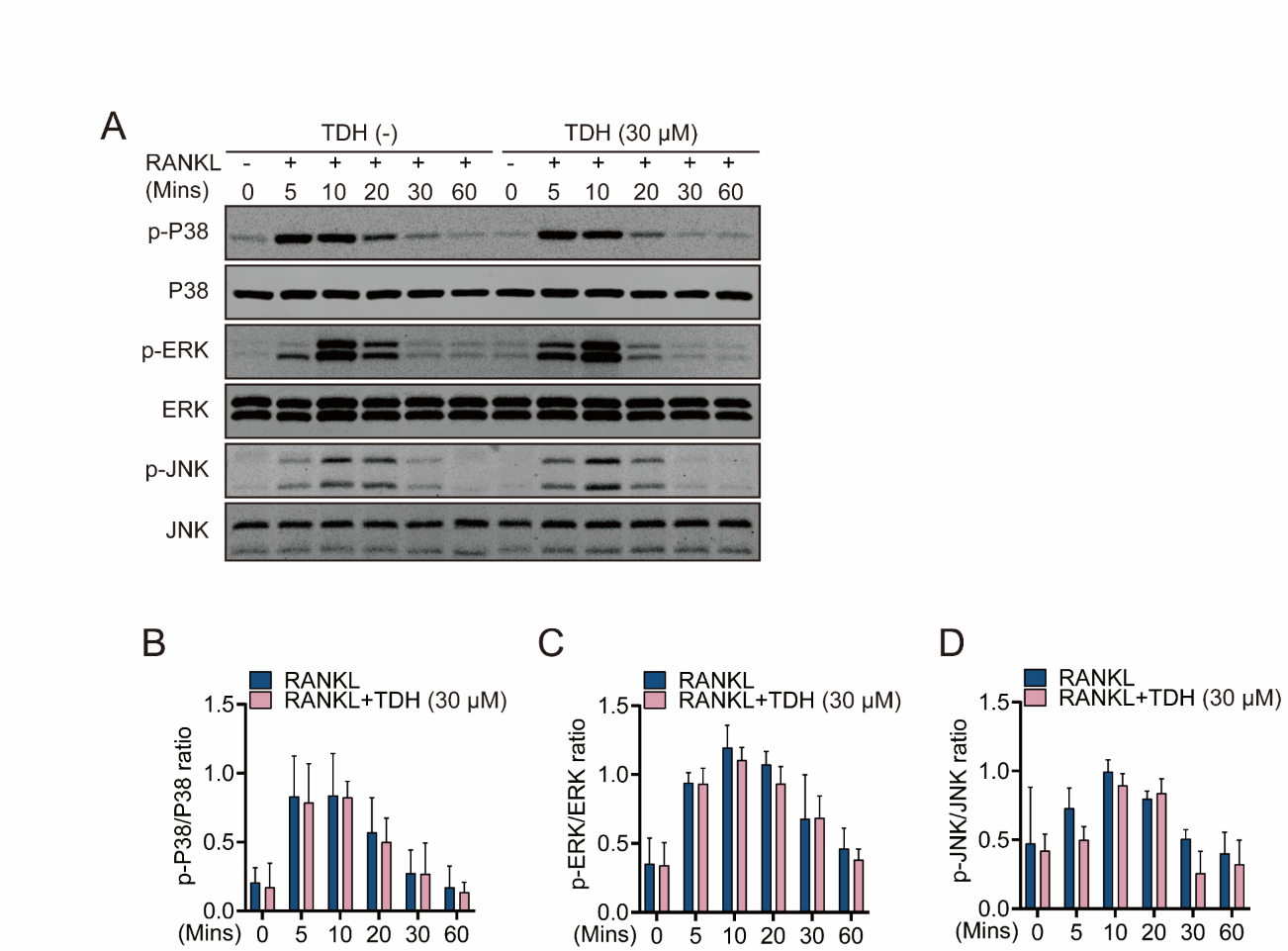


Supplementary Fig 2: TDH does not suppress MAPK pathway during osteoclastogenesis. (A) The protein expression levels of P38, ERK, JNK and their phosphorylated forms were measured by Western blot. (B-D) The ratio of p-P38/P38, p-ERK/ERK, p-JNK/JNK (n=5). All statistical histograms were expressed as means and standard deviations. * p < 0.05, ** p < 0.01, *** p < 0.001. TDH: tenacissoside H, RANKL: receptor activator of the nuclear factor‐κB ligand, MAPK: mitogen-activated protein kinases.
